# Editorial: Interactions of food components: structural changes and effects on nutritional value

**DOI:** 10.3389/fnut.2024.1354064

**Published:** 2024-01-24

**Authors:** Tiantian Zhao, Jianan Zhang, Qingrong Huang, Lanyue Zhang

**Affiliations:** ^1^Key Laboratory of Functional Foods, Guangdong Key Laboratory of Agricultural Products Processing, Ministry of Agriculture and Rural Affairs, Sericulture and Agri-Food Research Institute Guangdong Academy of Agricultural Sciences, Guangzhou, Guangdong, China; ^2^Department of Food Science, Rutgers University, New Brunswick, NJ, United States; ^3^School of Food Science and Engineering, South China University of Technology, Guangzhou, China; ^4^Guangdong Provincial Key Laboratory of Plant Resources Biorefinery, School of Biomedical and Pharmaceutical Sciences, Guangdong University of Technology, Guangzhou, China

**Keywords:** food component, interaction, food nutrition, food quality, bioactivity

In the realm of food science and nutrition, the intricate interplay of food components such as proteins, lipids, carbohydrates, polyphenols, and trace elements offers a rich tapestry of nutritional and flavor profiles. When consumed, these components engage in a dynamic interplay of chemical and physical interactions, profoundly altering their structure and, consequently, their nutritional value and bioavailability. Understanding these interactions is crucial for a comprehensive grasp of a food item's nutritional makeup and offers valuable guidance for healthy eating.

The rapidly evolving field of functional foods and nutritional science, spurred by increasing health consciousness and the challenges posed by modern dietary trends, has seen notable advancements. This editorial brings together five pivotal studies that focus on the interactions between food components in functional foods. Collectively, these studies deepen our understanding of the complexities inherent in food science and open new avenues for future food development and nutritional interventions.

Food component interactions significantly influence the quality, nutritional value, and sensory attributes of food. These interactions, occurring among proteins, carbohydrates, lipids, and other minor components, lead to modifications in food texture, stability, and nutrient bioavailability.

Firstly, a study on high amylose wheat (HAW) revealed its unique dough structure and its relationship with the properties of gluten and starch. In Xinong 836, a variety with high amylose content, the superior gluten structure, higher B-type starch content, and lower starch crystallinity and enthalpy synergistically improve the interaction between gluten and starch in the dough. The presence of a higher proportion of small starch granules in this variety appears to contribute to a denser dough structure, resulting in enhanced dough properties. These findings suggest that while superior gluten structure is fundamental, the specific characteristics of HAW starch, particularly the abundance of small starch granules, are crucial for optimizing gluten-starch interactions and achieving better dough quality (Li et al.).

Subsequently, we reviewed the incorporation of coarse cereals in Chinese Steamed Breads (CSBs) and their contribution to nutritional enhancement. The authors also proposed that in addition to investigating the mechanisms underlying quality changes in CSBs, it is imperative to further explore their health benefits. This includes examining factors that influence the expected glycemic index (GI) of these breads and assessing the impact of their prolonged consumption on conditions such as diabetes, hyperglycemia, and hypertension. Such research is pivotal in potentially positioning coarse cereals CSBs as a beneficial staple food option for individuals with impaired health (Yang and Wang).

Another study elucidates how water content and specific heating conditions influence the formation of starch-lipid complexes, particularly affecting their structure and digestibility. The authors observed that higher water content, combined with a two-step heating process (initially at 100°C for 10 min followed by 90°C for 60 min), fosters the formation of a greater number of complexes with improved short-range order. This heating regimen is especially conducive to the formation of type II complexes, whose quantity escalates with increased water content. Interestingly, while the overall digestion rate of WS-GMP complexes diminishes marginally with higher water content, a significant reduction in starch digestion is noted upon additional heating at 90°C for 60 min. It appears that the digestibility variations in WS-GMP complexes are predominantly influenced by the structural order induced by the heating conditions, rather than by the quantity of complexes formed. These findings offer valuable insights into the formation dynamics of starch-lipid complexes, highlighting their potential for modulating the functional and nutritional properties of starchy foods (Jiao et al.).

Apart from these, the bioactivity changes induced by the interactions should also be taken into account for human wellness. One research within our topic provides a comprehensive understanding of the potential health benefits of soluble dietary fibers (SDFs), particularly in terms of their cholesterol-lowering capabilities. The increasing importance of developing new, safe, and natural functional foods for blood lipid control is underscored, especially in light of recent regulatory changes such as EU Commission regulation 2022/860. The findings are part of an ongoing systematic investigation into the binding abilities of SDFs from various natural sources to selected bile salts, using isothermal titration calorimetry (ITC). This research aims to illuminate the mechanisms behind the cholesterol-lowering activity of these fibers. It indicates no specific interaction between certain gums and bile salts, suggesting alternative mechanisms for cholesterol-lowering effects, potentially related to changes in solution viscosity (Massa et al.).

Furthermore, a study focusing on oxidative stress induced by free radicals and the protective role of phytochemicals emphasizes the potential benefits of the anthocyanin and gingerol combination in cellular antioxidant responses. This research is significant in understanding how food components can work synergistically to enhance health benefits. In conclusion, the synergistic effects of anthocyanins and gingerols (Ac-G) formulations, demonstrated through their enhanced antioxidant and cytoprotective properties, represent a promising health avenue for consumers, health professionals, and the food industry. Incorporating these antioxidants into the diet or as supplements could bolster the body's endogenous systems, aiding in cellular protection against oxidative damage. This study has delved into cell-based assays to investigate biomarkers related to antioxidant activity and redox pro-survival pathways, offering insights that could support the supplemental use of such combinations (Abdurrahim et al.).

In conclusion, the research presented in this editorial underscores the importance of understanding the complex interactions between food components. These interactions can significantly impact the nutritional value, health benefits, and overall quality of food. As we continue to explore these interactions, we can expect to see the development of new, innovative food products that offer enhanced nutritional and health benefits. This will not only benefit consumers but also provide new opportunities for the food industry and health professionals. The future of food science and nutritional research looks bright.

## Author contributions

TZ: Conceptualization, Investigation, Resources, Supervision, Validation, Writing – original draft, Writing – review & editing. JZ: Writing – review & editing. QH: Writing – review & editing. LZ: Writing – review & editing.

